# Preliminary feasibility assessment of a targeted, pharmacist-led intervention for older adults with polypharmacy: a mixed-methods study

**DOI:** 10.1007/s11096-024-01740-y

**Published:** 2024-05-16

**Authors:** Lisheng Liu, Bernadette Brokenshire, Deborah Davies, Jeff Harrison

**Affiliations:** 1 School of Pharmacy, Faculty of Medical and Health Sciences, The University of Auckland, Private Bag 92019, Auckland, 1142 New Zealand; 2 Primary, Public and Community Health, Te Whatu Ora MidCentral District, Palmerston North, New Zealand

**Keywords:** Aged, Feasibility studies, Geriatrics, Inappropriate prescribing, Pharmacists, Polypharmacy

## Abstract

**Background:**

Polypharmacy is associated with the prescription of inappropriate medications and avoidable medication-related harm. A novel pharmacist-led intervention aims to identify and resolve inappropriate medication prescriptions in older adults with polypharmacy.

**Aim:**

To conduct a preliminary feasibility assessment of the intervention in primary care, testing whether specific components of the intervention procedures and processes can be executed as intended.

**Method:**

The mixed-methods study was approved by the New Zealand Health and Disability Ethics Committees and public health agency. Patients from a New Zealand general practice clinic were recruited over 4 weeks to receive the intervention. The preliminary feasibility assessment included measures of intervention delivery, patient-reported outcome measures, and perspectives from ten patients and six clinicians. Data were analysed quantitatively and qualitatively to determine if a full-scale intervention trial is warranted. The study's progression criteria were based on established research and guided the decision-making process.

**Results:**

The intervention met the study's progression criteria, including patient recruitment, retention, and adherence to the intervention procedures. However, several modifications were identified, including: (1) enhancing patient recruitment, (2) conducting a preliminary meeting between the patient and pharmacist, (3) supporting pharmacists in maintaining a patient-centred approach, (4) reviewing the choice of patient-reported outcome measure, (5) extending the 8-week follow-up period, (6) allocating more time for pharmacists to conduct the intervention.

**Conclusion:**

The study found the intervention feasible; however, additional development is required before progressing to a full-scale trial. This intervention has the potential to effectively reduce medication-related harm and improve outcomes for older adults with polypharmacy.

**Trial registration number:**

ACTRN12621000268842 Date registered: 11/03/2021

**Supplementary Information:**

The online version contains supplementary material available at 10.1007/s11096-024-01740-y.

## Impact statements

- A pharmacist-led intervention was evaluated in a primary care setting, demonstrating its feasibility. The findings suggest a potentially promising strategy to address problematic polypharmacy in older adults, paving the way for a comprehensive full-scale trial evaluation.
- The groundwork for a refined intervention has been set, which has the potential to decrease inappropriate medication prescribing, lower medication-related harm, and improve the quality of care for older adults.

## Introduction

Polypharmacy, which is the concomitant prescribing of multiple medications for patients, poses challenges for healthcare systems worldwide. Polypharmacy is driven by an ageing population, broader preventive treatments, and increasing comorbidities (the presence of multiple medical conditions) [1–3].

Traditionally, numerical thresholds have been used to define polypharmacy, with thresholds ranging from two or more to 11 or more being reported in the literature [4]. However, it is essential to note that more medications is not necessarily harmful in every instance. For example, in Payne et al.'s study, the risk of unplanned hospital admission for patients with comorbidities taking four to six medications was similar to those taking one to three (odds ratio 1.00; 95% confidence interval 0.88–1.14) [5].

Polypharmacy can be appropriate when prescribing is evidence-based for patients with comorbidities [1]. For instance, after a transient ischaemic attack or ischaemic stroke, combination treatment with multiple medications is often beneficial [6]. However, polypharmacy becomes problematic when potentially inappropriate medications (PIMs) are prescribed, where the medication harm outweighs the benefits, if the medication is no longer indicated, or if adverse medication interactions and events occur [1, 7].

Problematic polypharmacy is a particular concern for older adults due to their increased likelihood of accumulating comorbidities [1], as well as age-related physiological changes that heighten their vulnerability to medication-related harm [8]. Additionally, problematic polypharmacy can increase medication burden, impacting social and functional activities [9].

Explicit criteria have been used to identify and measure problematic polypharmacy. Explicit criteria contain a catalogue of PIMs drawn from literature and expert consensus. Examples of such lists of PIMs that are important to avoid or which should be used cautiously in older adults due to their potential for adverse outcomes include the international Beers Criteria [10] and STOPP/START Criteria [11], as well as country-specific criteria such as the New Zealand Criteria, which list PIM indicators that New Zealand healthcare experts recommend for formal review [12].

Another area of interest involves leveraging information technology to manage problematic polypharmacy. In 2023, Liu et al. introduced PolyScan, a tool to help clinicians identify older adults with polypharmacy and PIMs for intervention. PolyScan demonstrated strong performance, achieving 100% sensitivity, specificity, and positive and negative predictive values to screen for patients who require further review [13].

While explicit criteria and PolyScan have been useful for identifying and measuring problematic polypharmacy, these approaches cannot tailor medication therapy to individual patient characteristics and preferences. The researchers in this study emphasised the necessity for an effective intervention for older adults with problematic polypharmacy, considering each patient’s unique treatment priorities. To meet this need, a novel pharmacist-led intervention was developed for primary healthcare. Its aims to optimise medication use and reduce PIMs for older adults with problematic polypharmacy. This intervention combines the PolyScan tool with pharmacist-led educational outreach and medication review.

### Aim

In this study, the aim was to assess the preliminary feasibility of implementing the intervention within a general practice clinic. Specific components of the intervention procedures and processes were tested, and insights from patients and clinicians were gathered. The goal was to ascertain if a full-scale clinical trial of the intervention is warranted.

### Ethics approval

The study adhered to the Declaration of Helsinki principles and received approval from the New Zealand Health and Disability Ethics Committees (reference number: 20/STH/238 date: 12/01/2021) and the New Zealand public health agency, Te Whatu Ora Te Pae Hauora o Ruahine o Tararua (reference number: 2021.01.021 date: 20/04/2021).

## Method

The design of this mixed-method study was informed by the Medical Research Council framework for developing and evaluating interventions [14].

### The PolyScan information technology tool

PolyScan uses 21 PIM indicators from the New Zealand Criteria and is programmed to search hospital and emergency department records, as well as subsidised medication dispensing information from New Zealand pharmacies [13]. It focuses on older adults aged 65 years and over taking 11 or more New Zealand subsidised medications, listed within the New Zealand Pharmaceutical Schedule [15].

PolyScan determines the presence of each PIM indicator for each patient and prioritises patients based on the number of indicators they meet. PolyScan provides outcome data at various levels of aggregation. The tool identifies the common PIM indicators within each clinic, the patients under each prescriber's care with a PIM indicator, and the specific indicator, prescriber, and dispensing pharmacy for patients with PIM indicators.

### Study population

#### Recruitment of general practice clinic

Patients were recruited from a New Zealand general practice clinic over a 4-week period from May to June 2021. In New Zealand, general practice clinics serve as central healthcare hubs for a diverse population of older adults, offering services such as chronic disease management, prescription of medications, and referral to other healthcare professionals [16]. The researcher (LL) met with the clinic to describe the study and obtain consent from the chief executive officer, who signed a consent form to participate.

#### Recruitment of pharmacist

The pharmacist delivering the intervention was required to have possess: 1) a current New Zealand pharmacist Annual Practicing Certificate, 2) a postgraduate university qualification in clinical pharmacy, and 3) experience practising within general practice clinics.

#### Recruitment of patients

PolyScan was used to screen the general practice clinic's enrolled population for potentially eligible patients, who were then contacted by the clinic. As discussed, PolyScan identified patients aged 65 years or older, taking 11 or more New Zealand subsidised medications, and with PIM indicators. The selection of older adults taking 11 or more medications as an inclusion criterion was based on a combination of clinical relevance and alignment with outputs from the PolyScan tool. This threshold was intended to identify individuals with particularly complex medication management, significant medication burden, and at risk of medication-related harm.

For interested patients, convenience sampling included those who were: (1) aged 65 years or older, taking 11 or more medications and PIMs, as identified by PolyScan, (2) enrolled in the participating clinic, (3) able to provide informed consent. Patients not meeting these conditions were excluded. Eligible patients were enrolled in the study by the researcher (LL), who provided them with a participant information sheet and a consent form to sign before participating. The patients participating in the study ranged in age from 70 to 88 years. They comprised individuals of New Zealand European (n = 5) and New Zealand Māori ethnicity (n = 5). The majority were female (n = 8) as opposed to male (n = 2). The number of medications they took ranged between 11 to 13 daily.

### The pharmacist-led intervention

**Fig. 1:**
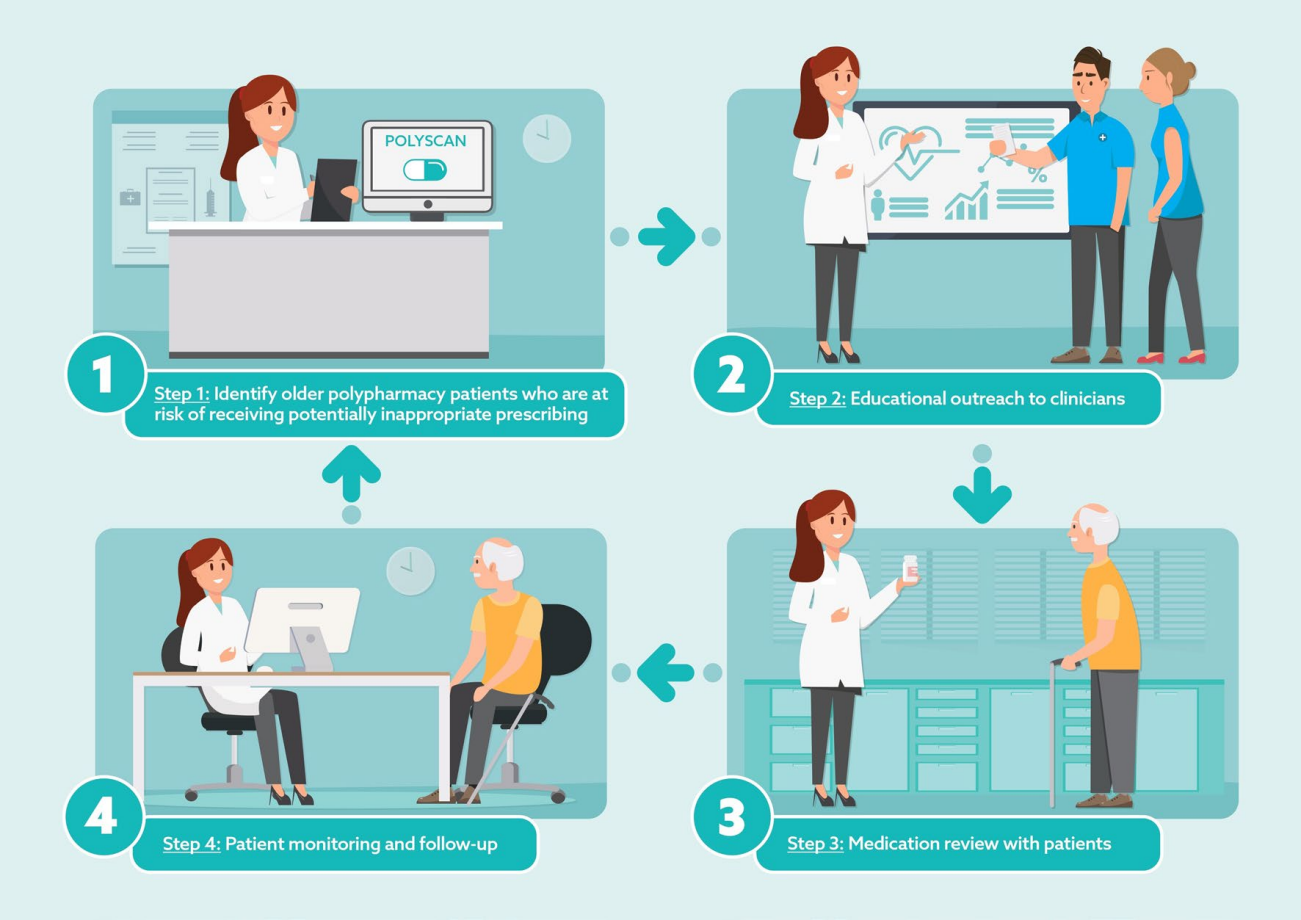
Procedures of the pharmacist-led intervention

Figure 1 outlines the intervention procedures, including patient identification, educational outreach, medication review, and follow-up.

PolyScan was used to identify older adults with polypharmacy and PIMs within the general practice clinic. The pharmacist then met with the general practice clinicians to: (1) discuss the outcomes generated by PolyScan, (2) provide education on problematic polypharmacy and rational medication use guidelines, (3) develop a plan to conduct medication reviews for patients.

The medication review involves the pharmacist conducting a Medication Therapy Assessment (MTA). In New Zealand, MTA is a medication management service provided by clinically experienced pharmacists within multidisciplinary healthcare teams. MTAs are "a systematic, patient-centred clinical assessment of all medicines currently taken by a patient" [17]. The pharmacist was allocated 30 min to meet with patients at the clinic or their homes to conduct each MTA. See Online Resource 1 for details of the MTA protocol.

Lastly, the pharmacist monitored patient medication response, including efficacy and safety, and followed up with the patient, their general practitioner, and other relevant healthcare team members.

### The preliminary feasibility assessment

Table 1 outlines the measures used to assess if specific intervention procedures and processes could be delivered as intended. These measures were developed based on Thabane et al.'s guidelines for feasibility studies [18]. A patient-reported outcome measure (PROM) as well as patient and clinician perspectives were also examined.

**Table 1:** Preliminary feasibility assessment measures

Assessment measure	Data type	Data collection and analysis procedure	Data collection time-point
Patient recruitment rate	Quantitative	Number of consented patients compared to the number of eligible patients*, as identified by PolyScan	1 Month post-medication reviews
Inclusion and exclusion criteria for participation	Quantitative	Number of referrals from the clinic compared to the number of eligible patients after screening Reasons for patient exclusions were identified from study field notes	1 Month post-medication reviews
Success and refusal rates to participate	Quantitative	Number of referred eligible patients compared to the number of patients who agree to participate Reasons for patients declining or barriers to participation were assessed via study field notes	1 Month post-medication reviews
Patient adherence to the study protocol	Quantitative	Number of patients who did not complete the medication review and LMQ-3	After each medication review and at 8-week follow-up
	Qualitative	Reasons for non-adherence to the study protocol were analysed via thematic analysis	
Time taken to complete the intervention	Quantitative	Time taken to complete the medication review and LMQ-3	After each medication review and at 8-week follow-up
Patient retention rate	Quantitative	Number of signed CFs compared to the number of patients requesting withdrawal Reasons for withdrawal were analysed via study field notes	At 8-week follow-up
Efficacy of the PolyScan tool for data collection and management	Quantitative	Patients* identified by PolyScan were compared against a manual review for 300 individuals aged 65 or older	At study commencement
Patient understanding of the intervention	Qualitative	Patient questions about the intervention were analysed via thematic analysis	After each medication review and at 8-week follow-up
Patient ability to complete the intervention and questionnaire	Qualitative	Patient challenges with the intervention and the LMQ-3 were analysed via thematic analysis	After each medication review and at 8-week follow-up
Appropriateness of the intervention location	Qualitative	Patient issues with the space for delivering the intervention were analysed via thematic analysis	After each medication review and at 8-week follow-up
Acceptability of the intervention for clinicians	Quantitative	ATCI-GP questionnaire scores were calculated by adding individual item scores and dividing by the number of items to generate a score out of five	At 8-week follow-up
Intervention completion	Quantitative	Number of pharmacist recommendations implemented by GPs were collected using clinic records	At 8-week follow-up
Patient-reported outcomes from the intervention	Quantitative	LMQ-3 scores were calculated by adding scores from each questionnaire item	After each medication review and at 8-week follow-up
Patient satisfaction with the intervention	Qualitative	Patient satisfaction with the intervention was analysed via thematic analysis	After each medication review and at 8-week follow-up

*ATCI-GP*, Attitudes towards collaboration instruments for general practitioners questionnaire; *CF*, Consent form; *GP*, General practitioner; *LMQ-3*, Living with medicines questionnaire version 3

^*^Older adults aged 65 years and over, taking 11 or more medications daily, and with potentially inappropriate medications

Following each medication review, the researcher (LL) met with patients either at the clinic or their homes for a healthcare assessment. Fifteen minutes were allocated for them to complete the Living with Medicines Questionnaire version 3 (LMQ-3), a PROM questionnaire evaluating their health and medication use [19]. After 8 weeks, the researcher met with patients again for a follow-up LMQ-3.

The LMQ-3 consists of 41 self-administered items for patients, rated on a five-point Likert scale from 'strongly agree' to 'strongly disagree', and grouped into eight domains. The scores within each domain generate a total score, where higher scores indicate a greater medication burden [19]. Additionally, the LMQ-3 includes a visual analogue scale allowing patients to rate their overall perceived medication burden from 'no burden at all' to 'extremely burdensome' [19]. The LMQ-3 was selected for this study due to its use in evaluating other interventions for older adults with polypharmacy [20] and its adaptation for the New Zealand population [21].

To gather patient perspectives, the researcher (LL) conducted interviews with patients at the clinic or their homes following each medication review. 8 Weeks after each medication review, a follow-up interview was conducted to gather patients' perceptions of the intervention's outcomes. A semi-structured interview guide was developed based on Beyene et al.'s research [22]. See Online Resource 2 for the interview questions.

To gather clinician perspectives, 8 weeks after completing the medication reviews, the clinic's general practitioners anonymously completed the Attitudes Towards Collaboration Instruments for General Practitioners questionnaire [23]. The questionnaire consists of 13 self-administered items, scored using a five-point Likert scale, where higher score reflect more positive attitudes towards pharmacist collaboration [23].

### Data analysis

Quantitative data were analysed to assess the study's progression criteria, which determined whether to proceed to a full-scale trial of the intervention (see Table 2). The criteria were developed based on research by Rankin et al. and Avery et al. [24, 25]. Qualitative data were analysed using thematic analysis to explore patient perspectives, following guidelines from Nowell et al. [26]. See Online Resource 3 for the thematic analysis protocol.

**Table 2:** Study progression criteria

Assessment measure	Stop	Amend	Go
Patient recruitment	≤ Four patients recruited in the 4-week recruitment period	Five to seven patients recruited in the 4-week recruitment period	≥ Eight patients recruited in the 4-week recruitment period
Patient retention rate	≤ 49.0% of patients retained at 8-week follow-up	50.0%–79.0% of patients retained at 8-week follow-up	≥ 80.0% of patients retained at 8-week follow-up
Patient adherence to the study protocol	≤ 49.0% of patients completed the medication review and LMQ-3 in its entirety	50.0–79.0% of patients completed the medication review and LMQ-3 in their entirety	≥ 80.0% of patients completed the medication review and LMQ-3 in their entirety
	*Description*		
Stop	A full-scale trial is not feasible if one or more assessment measures meets the 'Stop' criteria		
Amend	A full-scale trial is feasible with modifications to the protocol if the assessment measures meet the 'Amend' criteria		
Go	A full-scale trial is feasible without modifying the protocol or amendments to the protocol if the assessment measures meet the 'Go' criteria		

*LMQ-3*, Living with medicines questionnaire version 3

## Results

### Quantitative results

**Fig. 2:**
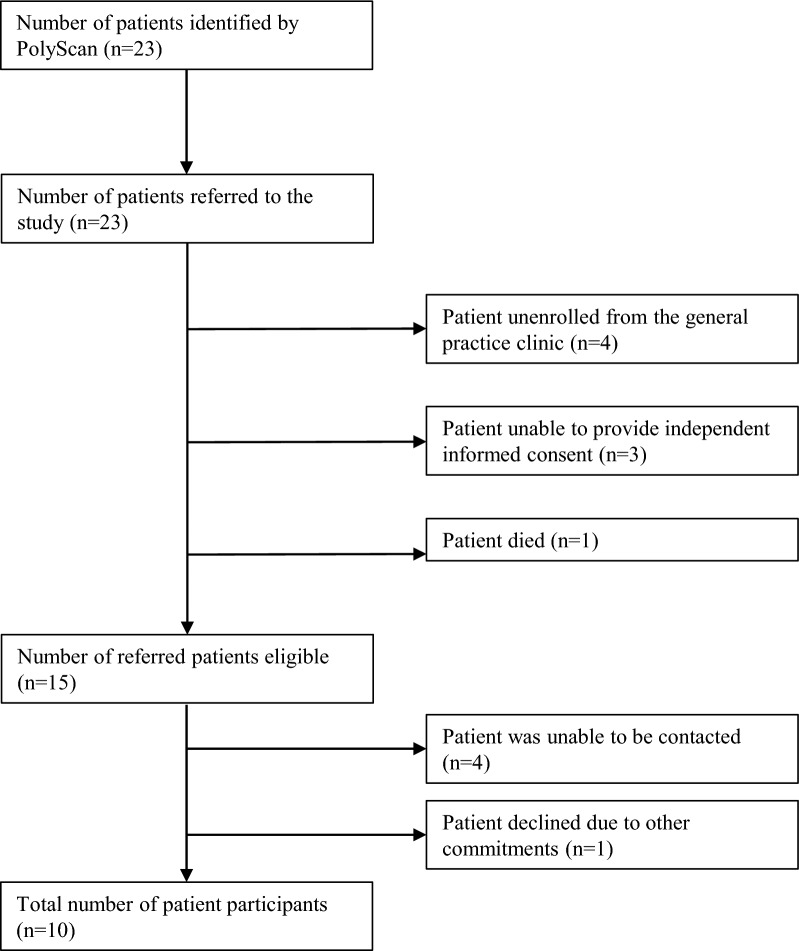
Summary flowchart of patient recruitment

During the May-to-June 2021 recruitment period, the clinic's enrolled population was 2,259 patients, with 215 patients aged 65 years or older. After PolyScan screening, 23 potentially eligible patients were referred by the clinic to the study (see Fig. 2). Of the 23 patients, 15 met the inclusion criteria, while eight were excluded. Reasons for exclusion included patients unenrollment from the clinic (n = 4), incapacity of independent informed consent (n = 3), and patient death (n = 1).

Of the 15 eligible referred patients who met the inclusion criteria, ten agreed to participate, while five were excluded. The reasons for exclusion included inability to be contacted (n = 4) or declining due to other commitments (n = 1). One patient withdrew from the study before the 8-week follow-up due to illness. The patient retention rate was 90 percent.

Regarding patient adherence, all patients completed the medication review and the initial LMQ-3. The median time to complete each medication review was 60 min. The median time to complete the LMQ-3 was 12 min for the initial and follow-up appointments.

Regarding the validity of PolyScan, as reported elsewhere by Liu et al., an assessment of PolyScan identified nine patients with polypharmacy and prescribed PIMs out of 300 older adults screened. Compared to a manual review, PolyScan achieved 100.0% sensitivity, specificity, and positive and negative predictive values [13].

Regarding clinician acceptability, all six general practitioners from the clinic completed the Attitudes Towards Collaboration Instruments for General Practitioners questionnaire. The summative scores ranged between four to five on a scale from one to five. See Online Resource 4 for results from the questionnaire.

Regarding intervention completion, the median number of pharmacist recommendations was two per patient. At 8-week follow-up, the median number of recommendations implemented by general practitioners was one per patient.

Patient medication burden was assessed using the LMQ-3 (see Table 3 for a summary of results). At the eight-week follow-up, the LMQ-3 score decreased for six patients, indicating an improvement in medication burden, and increased for three patients, indicating a worsening in medication burden. As for the LMQ-3 visual analogue scale, the score was reduced for five patients, indicating an improvement in medication burden, increased for two patients, indicating a worsening in medication burden, and remained unchanged for two patients. See Online Resource 5 for the complete results.

**Table 3:** Summary of living with medicines questionnaire version 3 (LMQ-3) results at the initial appointment and at 8-week follow-up

Patient (initial /follow-up appointment)	Total LMQ-3 score	Total LMQ-3 score categorised	LMQ-3 visual analogue scale score	LMQ-3 visual analogue scale score categorised
A—initial	89	Moderate burden	0	Minimal/no burden
A—follow-up	98	Moderate burden	1	Minimal/no burden
B—initial	87	Low burden	3.5	Minimal/no burden
B—follow-up	95	Moderate burden	0.5	Minimal/no burden
C—initial	115	High burden	5	Some degree of burden
C—follow-up	108	Moderate burden	2.5	Minimal/no burden
D—initial	72	Low burden	0	Minimal/no burden
D—follow-up	85	Low burden	0	Minimal/no burden
E—initial	103	Moderate burden	7	High degree of burden
E—follow-up	93	Moderate burden	1	Minimal/no burden
F—initial	96	Moderate burden	6.5	High degree of burden
F*—follow-up	N/A	N/A	N/A	N/A
G—initial	77	Low burden	4	Some degree of burden
G—follow-up	74	Low burden	5	Some degree of burden
H—initial	112	High burden	1	Minimal/no burden
H—follow-up	90	Moderate burden	0	Minimal/no burden
I—initial	118	High burden	8	High degree of burden
I—follow-up	90	Moderate burden	0	Minimal/no burden
J—initial	99	Moderate burden	0	Minimal/no burden
J—follow-up	79	Low burden	0	Minimal/no burden

Total LMQ-3 score categories: score 41–87 = low burden, score 88–110 = moderate burden, score > 110 = high burden

LMQ-3 visual analogue scale score categories: score 4.0 or lower = minimal/no burden, score 4.1–5.9 = some degree of burden, score 6.0 or higher = high degree of burden

^*^Patient F withdrew from the study before the 8-week follow-up

### Qualitative results

**Fig. 3:**
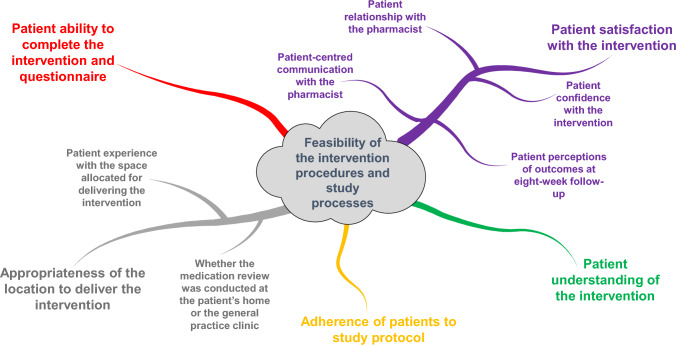
Concept map of themes and sub-themes

Five primary themes emerged from the patient interviews: (1) satisfaction with the intervention, (2) appropriateness of the location for delivering the intervention, (3) understanding of the intervention and questionnaire, (4) ability to complete the intervention and questionnaire, and (5) adherence to the study protocol. Figure 3 visually represents these themes and sub-themes.

#### Theme 1: patient satisfaction with the intervention

##### Subtheme 1: patient relationship with the pharmacist

Most patients developed a positive relationship with the pharmacist, feeling comfortable discussing their concerns, believing in the pharmacist’s genuine interest in their well-being, and trusting the pharmacist to help with decisions about their medications and healthcare.

One patient felt the pharmacist made assumptions without the necessary background understanding and that interpersonal trust could not be established. The patient suggested the pharmacist relay an awareness of her health background to build trust and collaborate on treatment plans.

##### Subtheme 2: patient-centred communication with the pharmacist

Most patients had positive experiences with the pharmacist's communication, feeling the pharmacist dedicated appropriate time to listen to health concerns, understood their health needs, explained things clearly and understandably, and involved them in decisions about their medications.

One patient expressed concerns that the pharmacist did not fully grasp her health needs. The patient felt the pharmacist used jargon and suggested that vulnerable people, such as those struggling to express themselves, might feel intimidated during the medication review. The patient suggested that to improve communication, the pharmacist should keep communication simple, slow down their speech, introduce less information initially, build more rapport, and ask patients why they are taking certain medications.

##### Sub-theme 3: patient confidence with the intervention

Most patients were confident in the pharmacist's services, satisfied with the time taken for medication review, and believed that others would benefit from the intervention.

Some patients felt that further engagement was necessary to establish an ongoing relationship with the pharmacist. The patient who previously expressed concerns suggested that an initial meeting to discuss the medication review process, the patient's health goals, and concerns would be beneficial.

##### Sub-theme 4: patient perceptions of outcomes at eight-week follow-up

Most patients were pleased with the intervention outcomes. Approximately half of patients found the outcomes helpful, and most did not report any problems or adverse effects.

One patient reported adverse outcomes. The patient believed that while the pharmacist acted appropriately, the outcomes were not helpful, leading to adverse effects. The patient suggested that it was important for the pharmacist to acknowledge her unique health situation and that medications can have multiple indications, which should be considered in consultation with her doctor.

#### Theme 2: appropriateness of the location to deliver the intervention

##### Sub-theme 1: whether the medication review was conducted at the patient's home or the general practice clinic

Most patients conducted the medication review at their homes, while one patient conducted the review at the clinic.

##### Sub-theme 2: Patient experience with the space allocated for delivering the intervention

Patients were satisfied with their medication review, whether it was provided at the clinic or home. They felt they could speak openly and privately in both environments. Most patients preferred face-to-face medication reviews over video or telephone calls.

#### Theme 3: patient understanding of the intervention

Most patients felt they understood the intervention, which was introduced clearly.

Some patients questioned the depth of the intervention and whether it might be perceived as solely about reducing medications. Patients suggested it would be helpful to clarify what is included in the medication review and reassure people that the intervention is not about reducing medications but to ensure that medications are appropriate.

#### Theme 4: patient ability to complete the intervention and questionnaire

Most patients did not find any aspects of the intervention to be difficult.

When asked if other older adults might find any aspects challenging, patients commented that some people might need help with the terminology or feel hesitant about coming forward to receive the intervention. Patients agreed that it was essential to engage with patients living alone or those more withdrawn, recommending the pharmacist initiate contact to develop a working relationship.

#### Theme 5: adherence of patients to study protocol

Most patients felt they could complete all the forms and the medication review. They did not find any questions confusing or inapplicable.

## Discussion

Managing patients with comorbidities can be challenging due to the complexities of patient health and medication regimens, as well as the time constraints placed on clinicians. This study seeks to support clinicians through a preliminary feasibility assessment of an intervention designed to optimise medication use and reduce PIMs for older adults with polypharmacy.

The intervention procedures and processes met the study's assessment measures, with patient recruitment, retention, and adherence to the intervention protocol meeting the progression criteria for a full-scale intervention evaluation. Additionally, patients found the intervention easy to understand, did not find the intervention challenging to complete, and were satisfied with the LMQ-3. Respondents of the Attitudes Towards Collaboration Instruments for General Practitioners questionnaire also reported positive attitudes toward collaboration with the pharmacist.

Although the study met the preliminary feasibility assessment measures, it also identified valuable insights, which suggest design modifications are needed before a full-scale trial.

Effective patient recruitment remains a crucial challenge, and eligibility criteria should be expanded to include patients unable to provide independent informed consent. Obtaining consent from a welfare guardian or enduring power of attorney ensures that patients unable to provide independent informed consent are not excluded from an intervention that could benefit their health. Furthermore, the inclusion criteria for this study was set for patients taking 11 or more medications. Given the variation in numerical definitions of polypharmacy [4], to expand the pool of eligible patients, it could be appropriate to lower the medication count required for inclusion in a future trial.

To enhance patient-pharmacist understanding and relationship building, a preliminary meeting should be arranged between the patient and the pharmacist to discuss the intervention and the patient's health. Additionally, it should be acknowledged that this intervention was not intended to and cannot replace opportunities to develop New Zealand indigenous Māori-led initiatives, such as Hikaka et al.'s medication intervention for Kaumatua (Māori elders) [27]. However, to ensure the quality of the intervention for Kaumatua, adopting Lacey et al.’s 'Hui Process' as a framework for the preliminary meeting and subsequent consultations could facilitate relationship building and ensure cultural safety [28].

Pharmacists delivering the intervention should receive training in consultation skills to support a patient-centred approach during the medication review. A future pharmacist training package may incorporate Wolters et al.’s patient-centred communication model [29] and Grimes and Barnett et al.'s consultation skills programme for improving communication, consultation, and health coaching skills [30].

Although patients expressed satisfaction with the LMQ-3, the PROM questionnaire lacks some essential information required to function as an outcome measure in a future clinical trial. A PROM selected for future use should provide data on sensitivity to change, minimal clinically important differences, and baseline score estimates. There is a lack of relevant data in the literature for the LMQ-3.

The 8-week follow-up period should be reconsidered, as some pharmacist recommendations were not yet implemented by general practitioners who reviewed patients on a 3-monthly prescription cycle. A longer follow-up period would allow practitioners more time to consider the pharmacist's recommendations and identify beneficial or hazardous effects that may only become evident long after the intervention [31, 32].

The time allocated for each medication review should be extended to 60 min. However, it is important to consider the implications of this increased time allocation for healthcare stakeholders and funders, as it may require additional resources and impact capacity. Nevertheless, it is worth noting that the medication review in this intervention was comprehensive and involved patients with complex medication regimens. Therefore, each review required more time than a standard consultation addressing a medical concern. Research has also established that investing time in a comprehensive medication review can lead to time and cost savings elsewhere by preventing adverse drug events, improving the quality of medication processes, and freeing up clinicians for other tasks [33–35].

Internationally, a variety of interventions have been developed to address problematic polypharmacy among older adults. In a Cochrane Review, 38 studies of relevant interventions were identified [36]. Among these, Basager et al. assessed a prescribing appropriateness criteria-set during medication reviews for older Australian adults taking five or more medications [37]. Campins et al. evaluated a medication assessment programme for community-dwelling older adults taking eight or more medications in Spain [38]. Muth et al. examined a complex intervention to improve medication appropriateness for older adults taking five or more medications in Germany [39].

Despite international efforts, no single intervention has been definitively proven to be the most effective. Additionally, many studies lack detailed information on intervention development and implementation, which is crucial for enhancing their efficacy and replicability across different settings [36]. This study contributes to the existing literature of interventions developed to address problematic polypharmacy in older adults. The study intervention is unique in its utilisation of PolyScan to identify older adults with polypharmacy and PIMs. The study also stands out from many existing studies with its systematic approach to intervention development and implementation.

A key strength of this study was the careful and deliberate method used to evaluate the intervention. The study employed clear measures to assess intervention procedures and processes, including quantitative and qualitative assessment measures and progression criteria. The study also had several constraints. Firstly, all patients received the intervention to test specific procedures and processes. As a result, aspects of the full-scale intervention, such as the recruitment of the control group, randomisation process, and allocation concealment, were beyond the scope of this study. Secondly, the study had a small sample size and a short follow-up period. However, the study was not designed to identify statistically significant long-term findings. Thirdly, the pharmacist and clinic were not blinded to the intervention, which could have influenced clinician behaviour and reported outcomes. Lastly, while established methods were used to analyse qualitative data, the interviewer's involvement in the intervention's development could have biased feedback. An independent interviewer might have reduced bias, but was not feasible due to budget and logistic constraints.

There is recognition that pharmacist-led interventions in primary care can have a positive impact on patient outcomes by reducing medication-related adverse effects, medication errors, and hospital admissions [40–42]. Through close collaboration with other healthcare professionals, pharmacists can contribute to improving patient safety and the quality of care provided by clinics [43]. Therefore, for general practice clinics seeking to enhance their services and improve patient outcomes, integrating pharmacists into their teams through this intervention is a promising initiative that warrants further evaluation.

For researchers, this study exemplifies the use of the Medical Research Council's best practice framework for developing and evaluating interventions [14]. Despite the framework's availability since 2008, limited interventions for older adults with polypharmacy have referenced it in their development [36]. Future research on similar interventions may benefit from adopting this framework to ensure that interventions are replicable, practical, and implementable across different settings.

Lastly, this study underscores the importance of feasibility testing intervention procedures and processes. Despite its necessity, research suggests that feasibility evaluations are often overlooked [44]. Researchers may consider using the mixed-method approach employed in this study to design similar feasibility studies in future research.

## Conclusion

This study indicates that implementing the intervention into general practice is feasible; however, modifications are necessary before proceeding to a full-scale clinical trial.

The next phase of this research will focus on developing a cluster-randomised controlled trial for the full-scale intervention. This trial will incorporate the changes identified in this study and provide details such as study duration, baseline data collection, definitive outcome measures, sample size, randomisation, blinding, and statistical methods. Economic and process evaluations will also be included to investigate cost-effectiveness and identify barriers to implementing pharmacist recommendations.

## Supplementary Information

Below is the link to the electronic supplementary material.

Supplementary file1 (PDF 268 KB)

Supplementary file2 (PDF 260 KB)

Supplementary file3 (PDF 257 KB)

Supplementary file4 (PDF 132 KB)

Supplementary file5 (PDF 316 KB)
