# Supplementary material for: Preliminary feasibility assessment of a targeted, pharmacist-led intervention for older adults with polypharmacy: a mixed-methods study
Source: Int J Clin Pharm. 2024 May 16;46(5):1102–13. doi: 10.1007/s11096-024-01740-y (PMC11399159; doi:10.1007/s11096-024-01740-y)
Supplement: Supplementary file 1 — Supplementary file1 (PDF 268 KB) [file 11096_2024_1740_MOESM1_ESM.pdf]

**Supplementary Information**

**Article title:** Preliminary feasibility assessment of a targeted, pharmacist-led intervention for older adults with polypharmacy: a mixed-methods study

**Journal name:** International Journal of Clinical Pharmacy

**Author names:** Lisheng Liu[1, 2], Bernadette Brokenshire[2], Deborah Davies[2], Jeff Harrison[1*]

**Affiliation:** [1] The University of Auckland, School of Pharmacy, Faculty of Medical and Health Sciences, Auckland, New Zealand. ORCID iD (Liu): 0000-0003-0280-4793, ORCID iD (Harrison): 0000-0001-8478-7469

[2] Te Whatu Ora, Primary, Public and Community Health, MidCentral District, Palmerston North, New Zealand.

*__Corresponding author:__ Email: jeff.harrison@auckland.ac.nz Postal: Private Bag 92019, Auckland 1142, New Zealand.

**Online Resource 1** Pharmacist protocol for conducting Medication Therapy Assessments

1. Gather patient health and medication history, lifestyle, immunisation status, and understand the patient's values and goals regarding their health and medications.

2. Evaluate patient characteristics, including cultural background, language barriers, literacy level, and education level, which may affect their health or medication outcomes.

3. Evaluate whether any patient-reported symptoms could be attributed to adverse effects or medication interactions.

4. Interpret, assess, and monitor patient laboratory results.

5. Assess, detect, and prioritise patient medication concerns, including:
   - Unnecessary medication therapy or inappropriate polypharmacy.
   - Inappropriate medications, dosage, or dosing regimen to achieve therapeutic objectives.
   - Adverse medication effects, contraindications, or interactions.
   - Poor medication adherence.
   - Untreated medical conditions.
   - Healthcare or medication access and cost.

6. Work with the patient to develop a tailored, mutually agreed plan to resolve any identified medication-related issues. Provide medication education, including improving medication adherence and understanding of treatment goals, so the patient is empowered to manage their medications and health.

7. Communicate recommendations to the patient's general practitioner to improve medication efficacy, resolve inappropriate polypharmacy or other identified medication-related problems, follow-up and monitoring. Recommendations are discussed with the general practitioner in person or through electronic messages within the practice management system. The general practitioner is free to accept or decline the recommendations.

8. Monitor, follow-up, and evaluate patient medication response, including efficacy and safety.
