# Supplementary material for: Preliminary feasibility assessment of a targeted, pharmacist-led intervention for older adults with polypharmacy: a mixed-methods study
Source: Int J Clin Pharm. 2024 May 16;46(5):1102–13. doi: 10.1007/s11096-024-01740-y (PMC11399159; doi:10.1007/s11096-024-01740-y)
Supplement: Supplementary file 2 — Supplementary file2 (PDF 260 KB) [file 11096_2024_1740_MOESM2_ESM.pdf]

Supplementary Information

Article title: Preliminary feasibility assessment of a targeted, pharmacist-led intervention for older adults with polypharmacy: a mixed-methods study

Journal name: International Journal of Clinical Pharmacy

Author names: Lisheng Liu[1, 2], Bernadette Brokenshire[2], Deborah Davies[2], Jeff Harrison[1*]

Affiliation: [1] The University of Auckland, School of Pharmacy, Faculty of Medical and Health Sciences, Auckland, New Zealand. ORCID iD (Liu): 0000-0003-0280-4793, ORCID iD (Harrison): 0000-0001-8478-7469

[2] Te Whatu Ora, Primary, Public and Community Health, MidCentral District, Palmerston North, New Zealand.

*__Corresponding author:__ Email: jeff.harrison@auckland.ac.nz Postal: Private Bag 92019, Auckland 1142, New Zealand.

Online Resource 2 Interview guide

Orienting question:
"As you know, we're introducing a new way of making sure people's medicines are both working for them and safe to be used together. This service will particularly focus on people taking lots of medicines or unusual combinations of medicines. What I want to talk to do today is talk to you about your experience of this service. What I'm hoping you can do is help us make this work well from a patient's point of view".

Participant satisfaction with the intervention
Dimension 1: Patient-pharmacist relationship
Main question:
"How did you feel about your relationship with the pharmacist?"
Follow-up questions:
"Did you feel the pharmacist had expressed a genuine interest in your wellbeing?"
"Did you feel comfortable discussing your concerns with the pharmacist?"
"Did you feel you could trust the pharmacist to help you make decisions about your medicines or other care?"

Dimension 2: Patient-centred communication
Main question:
"How did you feel about communicating with the pharmacist?"
Follow- up questions:
"Did you feel that the pharmacist took time to listen to your health concerns?"
"Did you feel the pharmacist understands your health needs?"
"Did the pharmacist explain things in a way you could clearly understand?"
"Did you feel the pharmacist involved you in making decisions about your medicines?"

Dimension 3: Confidence with service
Main question:
"I am also interested in understanding whether you felt confident with the services the pharmacist provided. Can you provide me with some of your thoughts?"
Follow-up questions:
"Were you satisfied with the time the pharmacist took with you to review your medicines?"
"Do you think other people would benefit from the service?"

At 8-week follow-up:
"What were the outcomes from the pharmacist consultation for you?"
"Were you happy with the outcomes of the consultation?"
"Do you feel the outcomes of the consultation helped you to get what you wanted out of your treatment?"
"Did you experience any problems or negative effects as a result of the consultation?"

Appropriateness of the location to deliver the intervention

Main question:
"Did you have your medication review at home or at the general practice?"

Main question:
"How did you feel about where this service was provided?"
Follow-up questions:
"Were you able to conveniently travel to the clinic to see the pharmacist?"
"Were you able to easily find the pharmacist for your appointment?"
"Were you able to talk to the pharmacist privately about your medicines and health?"

Main question:
"We always tend to think face-to-face is best for people, but do you think this service is something that could be done by phone or video-call?"
Follow-up question:
"Do you think it would be easier for people if this service was done in the home?"

Participant understanding of the intervention
Main question:
"Did you find it easy to understand what the service was for and why you'd been invited to the appointment?"
Follow-up question:
"Were there any things that weren't clear?"

Participant ability to complete the intervention and questionnaire
Main question:
"Can you tell me anything about either the organisation or the actual visit that you found difficult, or that were okay for you but you think other people in your situation might find hard?"

Adherence of participants to study protocol
Main question:
"Were you able to complete all of the forms and all of the visits?"
If not then follow-up question:
"Can you tell me a little bit more about that?"
"Did you feel the questions didn't apply to you, or were they confusing in any way?"

Closing question:
"Is there anything I should have asked but didn't, or anything else that you would like to add?"
