# Supplementary material for: Preliminary feasibility assessment of a targeted, pharmacist-led intervention for older adults with polypharmacy: a mixed-methods study
Source: Int J Clin Pharm. 2024 May 16;46(5):1102–13. doi: 10.1007/s11096-024-01740-y (PMC11399159; doi:10.1007/s11096-024-01740-y)
Supplement: Supplementary file 3 — Supplementary file3 (PDF 257 KB) [file 11096_2024_1740_MOESM3_ESM.pdf]

Supplementary Information

Article title: Preliminary feasibility assessment of a targeted, pharmacist-led intervention for older adults with polypharmacy: a mixed-methods study

Journal name: International Journal of Clinical Pharmacy

Author names: Lisheng Liu[1, 2], Bernadette Brokenshire[2], Deborah Davies[2], Jeff Harrison[1*]

Affiliation: [1] The University of Auckland, School of Pharmacy, Faculty of Medical and Health Sciences, Auckland, New Zealand. ORCID iD (Liu): 0000-0003-0280-4793, ORCID iD (Harrison): 0000-0001-8478-7469

[2] Te Whatu Ora, Primary, Public and Community Health, MidCentral District, Palmerston North, New Zealand.

***Corresponding author:** Email: jeff.harrison@auckland.ac.nz Postal: Private Bag 92019, Auckland 1142, New Zealand.

Online Resource 3 Protocol for conducting thematic analysis

1. Patient interviews were audio-recorded and transcribed into textual transcript data by the author (LL). Microsoft® Excel® software was used for logging each patient's transcripts to their assigned unique code to identify the source of the data and the date it was collected. The transcripts were stored in a secure network, dated, and archived. The transcripts were analysed manually by the author (LL). The author (LL) was immersed in the data by actively reading through the entirety of transcripts multiple times to identify patterns and meanings before coding. The author (LL) journaled any theoretical or reflective thoughts about the data, including any ideas for coding.

2. Codes were developed through reflection and interaction with the data. The coding process systematically focused the raw unstructured transcripts into segments important for determining the success of the feasibility assessment measures. All transcripts were read to note ideas and sense of the entirety of the data. One patient transcript was selected, and its underlying topics were noted. After completing this process for several transcripts, a list of identified topics was assembled. The assembled topics were then abbreviated into codes, and transcript segments were coded accordingly. Subsequently, authors (LL, JH) scrutinised the coding and determined that data saturation was reached, as the same codes were consistently observed, and no new codes were identified.

3. The coded transcript extracts were catalogued into themes and sub-themes. Similar related topics (codes) were grouped and categorised into themes and sub-themes using the most descriptive wording. Transcript extracts were coded into as many themes and as many times as relevant. Any extracts from an interview question that revisited an earlier theme were catalogued within the appropriate earlier theme.

4. The author (LL) reviewed the codes from each subtheme to ensure accuracy and coherency with the data. New codes were to be inserted if a new topic was identified, and existing codes were removed if they were unnecessary or duplicated. Similarly, themes with insufficient data were removed, whilst other themes were broken into separate or new themes. Themes were not finalised until all transcript data was read and evaluated by the author (LL). Following the review, the author (LL) concluded that no refinements were required.

5. The author (LL) analysed the themes to identify the narrative expressed by each theme and how they connected to the overall question of feasibility. The author (LL) further scrutinised each theme to ensure themes were reflected across all patients and no aspects were omitted. Authors (LL, JH) also discussed the appropriateness of the themes. The consensus was reached that the themes, sub-themes, and coding were appropriate and sensible. The feedback was recorded as an audit trail.

6. All themes, including patient concordance and disagreement, were discussed when reporting the thematic analysis results.

7. Throughout the thematic analysis process, the author (LL) collected raw interview data, transcripts, field notes, and kept a reflexive journal that explained the rationale behind analytical decisions and the development of themes.
