# Supplementary material for: Preliminary feasibility assessment of a targeted, pharmacist-led intervention for older adults with polypharmacy: a mixed-methods study
Source: Int J Clin Pharm. 2024 May 16;46(5):1102–13. doi: 10.1007/s11096-024-01740-y (PMC11399159; doi:10.1007/s11096-024-01740-y)
Supplement: Supplementary file 4 — Supplementary file4 (PDF 132 KB) [file 11096_2024_1740_MOESM4_ESM.pdf]

Supplementary Information

Article title: Preliminary feasibility assessment of a targeted, pharmacist-led intervention for older adults with polypharmacy: a mixed-methods study

Journal name: International Journal of Clinical Pharmacy

Author names: Lisheng Liu[1, 2], Bernadette Brokenshire[2], Deborah Davies[2], Jeff Harrison[1*]

Affiliation: [1] The University of Auckland, School of Pharmacy, Faculty of Medical and Health Sciences, Auckland, New Zealand. ORCID iD (Liu): 0000-0003-0280-4793, ORCID iD (Harrison): 0000-0001-8478-7469

[2] Te Whatu Ora, Primary, Public and Community Health, MidCentral District, Palmerston North, New Zealand.

*__Corresponding author:__ Email: jeff.harrison@auckland.ac.nz Postal: Private Bag 92019, Auckland 1142, New Zealand.

Online Resource 4 Results from Attitudes Toward Collaboration Instrument for GPs questionnaire

Question number		General practitioner					
		**A**	**B**	**C**	**D**	**E**	**F**
1.	The professional communication between myself and the pharmacist is open and honest	4	5	5	5	5	4
2.	The pharmacist is open to working together with me on patients' medication management	4	5	5	5	5	5
3.	The pharmacist delivers high quality healthcare to patients	4	5	5	5	5	5
4.	The pharmacist has time to discuss with me matters relating to patients' medication regimens	4	5	5	5	5	4
5.	The pharmacist meets the professional expectations I have of him/her	4	5	5	5	5	5
6.	I trust the pharmacist's professional decisions	4	5	5	5	5	5
7.	The pharmacist actively addresses patients' medical concerns	4	5	5	5	5	4
8.	The pharmacist and I have mutual respect for one another on a professional level	4	5	5	5	5	4
9.	The pharmacist and I share common goals and objectives when caring for the patient	4	5	5	5	5	4
10.	My role and the pharmacist's role in patient care are clear	4	5	5	5	5	4
11.	I have confidence in the pharmacist's expertise in medicines and therapeutics	4	5	5	5	5	4
12.	The pharmacist has a role in assuring medication safety (for example, to identify drug interactions, adverse reactions, contraindications etc.)	4	5	5	5	5	4
13.	The pharmacist has a role in assuring medication effectiveness (for example, to ensure the patient receives the optimal drug at the optimal dose etc.)	4	5	5	5	5	4
**Summative scores**		4	5	5	5	5	4.3

Respondents were requested to indicate the extent to which they agree or disagree with the statements, where 1 = 'strongly disagree' and 5 = 'strongly agree'.
