# Supplementary material for: Preliminary feasibility assessment of a targeted, pharmacist-led intervention for older adults with polypharmacy: a mixed-methods study
Source: Int J Clin Pharm. 2024 May 16;46(5):1102–13. doi: 10.1007/s11096-024-01740-y (PMC11399159; doi:10.1007/s11096-024-01740-y)
Supplement: Supplementary file 5 — Supplementary file5 (PDF 316 KB) [file 11096_2024_1740_MOESM5_ESM.pdf]

Supplementary Information

Article title: Preliminary feasibility assessment of a targeted, pharmacist-led intervention for older adults with polypharmacy: a mixed-methods study

Journal name: International Journal of Clinical Pharmacy

Author names: Lisheng Liu[1, 2], Bernadette Brokenshire[2], Deborah Davies[2], Jeff Harrison[1*]

Affiliation: [1] The University of Auckland, School of Pharmacy, Faculty of Medical and Health Sciences, Auckland, New Zealand. ORCID iD (Liu): 0000-0003-0280-4793, ORCID iD (Harrison): 0000-0001-8478-7469

[2] Te Whatu Ora, Primary, Public and Community Health, MidCentral District, Palmerston North, New Zealand.

***Corresponding author:** Email: jeff.harrison@auckland.ac.nz Postal: Private Bag 92019, Auckland 1142, New Zealand.

Online Resource 5 Participant LMQ-3 results at initial appointment and at eight-week follow-up

Participant (initial /follow-up appointment)	LMQ-3 Domain 1	LMQ-3 Domain 2	LMQ-3 Domain 3	LMQ-3 Domain 4	LMQ-3 Domain 5	LMQ-3 Domain 6	LMQ-3 Domain 7	LMQ-3 Domain 8	Total LMQ-3 score	Total LMQ-3 score categorised
A - initial	9	12	11	5	13	15	10	14	89	Moderate burden
A – follow-up	8	13	11	8	10	18	18	12	98	Moderate burden
B – initial	7	16	5	7	17	13	11	11	87	Low burden
B – follow-up	10	14	6	8	13	17	15	12	95	Moderate burden
C – initial	9	21	11	11	14	25	15	9	115	High burden
C – follow-up	10	18	11	8	14	23	18	6	108	Moderate burden
D – initial	5	8	3	4	8	17	12	15	72	Low burden
D – follow-up	10	12	5	7	12	12	13	14	85	Low burden
E – initial	9	15	11	7	12	18	19	12	103	Moderate burden
E – follow-up	10	12	10	8	10	21	12	10	93	Moderate burden
F – initial	8	14	8	11	10	20	18	7	96	Moderate burden
F – follow-up	N/A	N/A	N/A	N/A	N/A	N/A	N/A	N/A	N/A	N/A
G – initial	6	12	6	11	9	17	8	8	77	Low burden
G – follow-up	5	17	3	5	8	20	8	8	74	Low burden
H – initial	10	20	9	12	14	21	19	7	112	High burden
H – follow-up	10	14	6	8	11	15	16	10	90	Moderate burden
I – initial	6	23	12	12	11	30	21	3	118	High burden
I – follow-up	5	16	6	8	10	19	17	9	90	Moderate burden
J – initial	14	19	7	8	10	15	14	12	99	Moderate burden
J – follow-up	9	10	5	6	11	13	10	15	79	Low burden

Total LMQ-3 score categories: score 41 – 87 = low burden, score 88 – 110 = moderate burden, score > 110 = high burden.
Abbreviations: LMQ-3, Living with Medicines Questionnaire version 3.

Participant LMQ-3 visual analogue scale results at initial appointment and eight-week follow-up

Participant (initial /follow-up appointment)	LMQ-3 visual analogue scale score	LMQ-3 visual analogue scale score categorised
A – initial	0	Minimal/no burden
A – follow-up	1	Minimal/no burden
B – initial	3.5	Minimal/no burden
B – follow-up	0.5	Minimal/no burden
C – initial	5	Some degree of burden
C – follow-up	2.5	Minimal/no burden
D – initial	0	Minimal/no burden
D – follow-up	0	Minimal/no burden
E – initial	7	High degree of burden
E – follow-up	1	Minimal/no burden
F – initial	6.5	High degree of burden
F – follow-up	N/A	N/A
G – initial	4	Some degree of burden
G – follow-up	5	Some degree of burden
H – initial	1	Minimal/no burden
H – follow-up	0	Minimal/no burden
I – initial	8	High degree of burden
I – follow-up	0	Minimal/no burden
J – initial	0	Minimal/no burden
J – follow – up	0	Minimal/no burden

LMQ-3 visual analogue scale score categories: score 4.0 or lower = minimal/no burden, score 4.1 to 5.9 = some degree of burden, score 6.0 or higher = high degree of burden.

Abbreviations: LMQ-3, Living with Medicines Questionnaire version 3.
